# Microscopy and Spectroscopy Techniques for Characterization of Polymeric Membranes

**DOI:** 10.3390/membranes10020033

**Published:** 2020-02-24

**Authors:** Yousef Alqaheem, Abdulaziz A. Alomair

**Affiliations:** Petroleum Research Center, Kuwait Institute for Scientific Research, Safat 13109, Kuwait; aomair@kisr.edu.kw

**Keywords:** gas-separation membrane, membrane characterization, surface analysis, crystal structure, functional groups, elemental composition

## Abstract

Polymeric membrane is a proven technology for water purification and wastewater treatment. The membrane is also commercialized for gas separation, mainly for carbon dioxide removal and hydrogen recovery. Characterization techniques are excellent tools for exploring the membrane structure and the chemical properties. This information can be then optimized to improve the membrane for better performance. In this paper, characterization techniques for studying the physical structure such as scanning electron microscopy (SEM), transmission electron microscopy (TEM), and atomic force microscopy (AFM) are discussed. Techniques for investigating the crystal structure such as X-ray diffraction (XRD), small-angle X-ray scattering (SAXS), and wide-angle X-ray scattering (WAXS) are also considered. Other tools for determining the functional groups such Fourier transform infrared spectroscopy (FTIR), Raman spectroscopy, and nuclear magnetic resonance (NMR) are reviewed. Methods for determining the elemental composition such as energy-dispersion X-ray spectroscopy (EDS), X-ray fluorescent (XRF), and X-ray photoelectron spectroscopy (XPS) are explored. The paper also gives general guidelines for sample preparation and data interpretation for each characterization technique.

## 1. Introduction

Characterization is an important field in material science. It refers to determining the physical and chemical properties of the material for a better understanding. This high level of knowledge can be then engineered to optimize the material performance. In membrane science, characterization techniques are widely used to confirm the quality and the purity of the prepared membranes. Furthermore, characterization techniques are powerful tools for interpreting the membrane performance and studying membrane degradation. Moreover, intensive research is carried out on preparing membranes from different materials to overcome the limitation in polymeric membranes. This limitation is due to the tradeoff between permeability and selectivity. This means that the produced gas can either have high flowrate or high purity [1]. This limitation was overcome by copolymerization, functionalization, or adding fillers [2]. Characterization techniques give valuable information on the interaction between the polymer and the fillers.

This paper reviews common spectroscopy techniques for the characterization of polymeric membranes. The techniques usually study the morphology data, crystal structure, functional groups, and chemical composition. For membrane morphology, scanning electron microscopy (SEM), transmission electron microscopy (TEM), and atomic force microscopy (AFM) are widely implemented. For studying the crystal structure and its shape and size, X-ray diffraction (XRD) is classically applied. Techniques such as small-angle X-ray scattering (SAXS) and wide-angle X-ray scattering (WAXS) can provide crystallography data with added information about the particle size and pore size distribution. Fourier-transform infrared (FTIR) spectroscopy, Raman spectroscopy, and nuclear magnetic resonance spectroscopy (NMR) are generally utilized for determining the functional groups. For measuring the chemical composition, energy-dispersion X-ray (EDS), X-ray fluorescent (XRF), and X-ray photoelectron spectroscopy (XPS) are commonly employed. In this paper, each technology is discussed in terms of operation, sample preparation, and limitations. The paper also gives general guidelines for interpreting the data for polymeric membranes.

## 2. Morphology Analysis

### 2.1. Scanning Electron Microscopy

Scanning electron microscopy (SEM) is one of the fundamental techniques for membrane characterization as it gives the morphology and topography data of the prepared membranes [3]. Furthermore, SEM can be used to determine the pore size in the case of a porous membrane [4]. For a dense membrane, SEM is used to measure the thickness of the selective layer for calculation of the permeability in Barrer [5].

**Figure 1:**
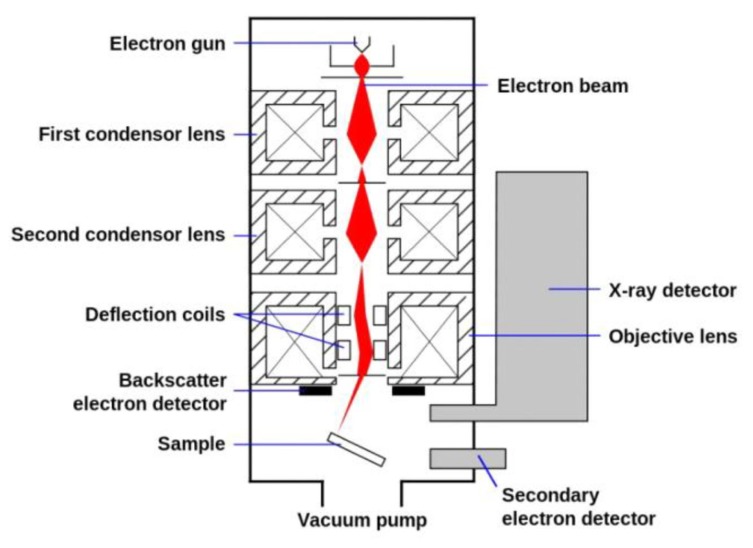
Components of SEM device for surface magnification of membranes [14].

SEM works similarly to an optical microscope but in SEM, electrons are used instead of light [6]. A beam of concentrated electrons will be sent to the sample and this will cause a release of secondary electrons [7]. After that, the electrons are collected by a detector and then analyzed to form an image as demonstrated in Figure 1. Compared to a light microscope, which gives a maximum magnification of 1500, the magnification in SEM can reach up to 1 million [8].

#### 2.1.1. Sample Preparation

Most of the membrane samples require preparation before using SEM. First, the samples should be solid and not exceed an area of 10 by 4 cm [9]. If the sample is larger, cutting it into smaller pieces is then required. Second, the samples should be electrically conductive in order to have good images [10]. Due to the weak conductivity in most polymers, the membrane surface needs to be coated with a conductive material such as gold or palladium [11]. Usually, the sample will be coated with a sputter-coater to form a thin film of gold of 10 nm [12]. In addition to improving the quality of the analyzed images, the coating also reduces the thermal damage on the sample due to charge build-up [13].

SEM can measure the thickness of the dense layer too by cutting the membrane and analyzing the cross-section area. Before coating the sample, it is advised to insert the sample in liquid nitrogen for a few minutes then cut it by a sharp blade. This will result in a better image and eliminates surface bend [15].

#### 2.1.2. Data Interpretation

**Figure 2:**
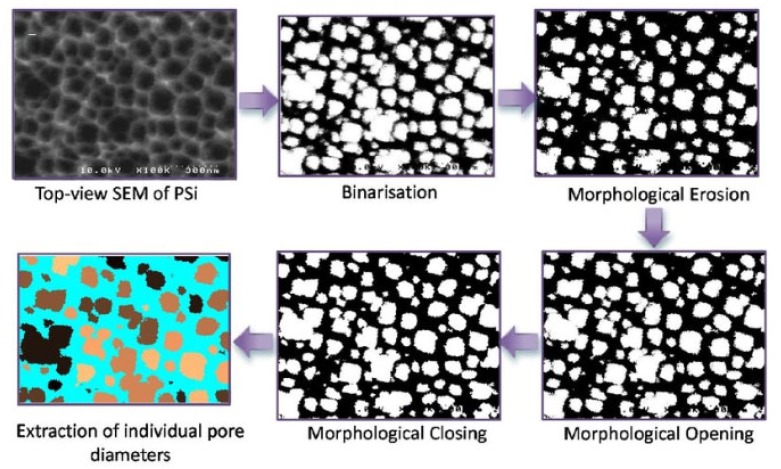
Image processing of SEM data to calculate the pore size profile of a silicon film using MATLAB [18].

Data in SEM is given in the form of a surface image. The membrane surface is expected to be free from defects such as large holes and cracks. For a porous membrane, the surface is expected to have a uniform structure of small holes. SEM can be used to calculate the average pore size by processing the image by a software such as MATLAB and the function “regionprops” as shown in Figure 2.

**Figure 3:**
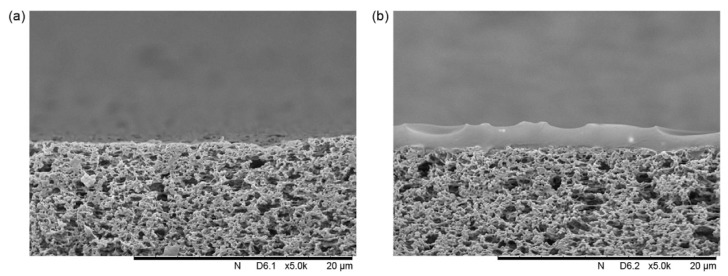
SEM image of: (**a**) Non-modified microfiltration membrane (MFFK-1), (**b**) composite poly(1-trimethylsilyl-1-propyne) (PTMSPM) with MFFK-1 support [17].

SEM is an excellent tool for analyzing composite membranes made from two or more polymers. Usually, a thin layer of the selective polymer is deposited over a porous support for better mechanical properties [16]. Examining the cross-section surface can give information about the structure of the two polymers. For example, Figure 3 shows SEM images of a composite membrane made from poly(1-trimethylsilyl-1-propyne) (PTMSP) over microfiltration membrane (MFFK-1). SEM was furtherly used to calculate the thickness of the PTMSP layer, which has an average value of 1.75 μm [17].

For polymeric gas separation membranes, the transport mechanism is mainly based on the solution-diffusion model, which states that the gas dissolves through the polymer and then diffuses.

**Figure 4:**
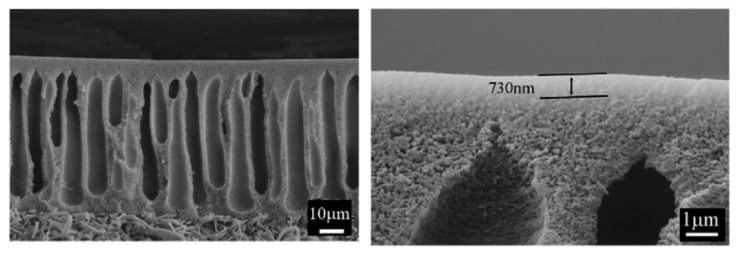
SEM image of the cross-section polyetherimide membrane showing porous and dense structures (**left**) and measurement of the dense layer (**right**) [19].

In these membranes, the surface should be fully dense and free from fully penetrating holes. However, by analyzing the cross-section surface, two layers of dense and porous structures are usually observed [5]. This is because during the membrane preparation, a solvent is used and then removed causing the formation of a porous structure as shown in Figure 4. To calculate the gas permeability in Barrer, the thickness of the dense layer is needed and this can be measured by SEM as demonstrated in Figure 4.

**Figure 5:**
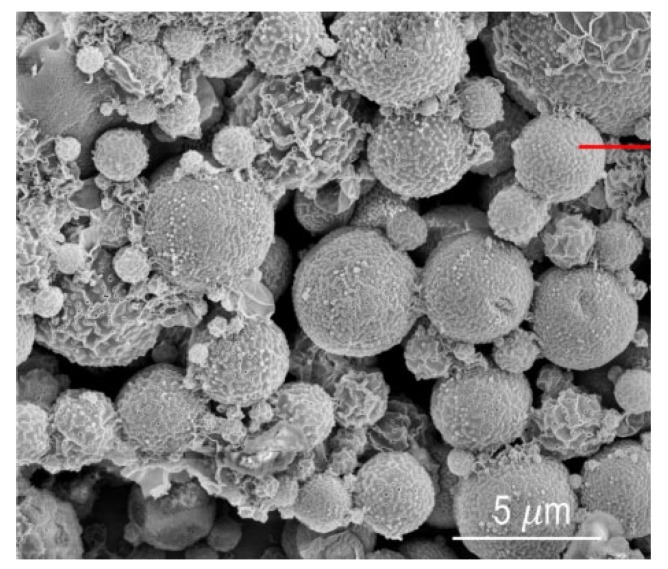
Particle size measurements of polysulfone (PS) and polyacrylic acid (PAA) co-polymer by SEM [21].

Copolymerization produces a polymer made from two or more monomers of different materials. This can be beneficial in improving the polymer characteristics such as mechanical properties, thermal stability, or gas transport [20]. Porous particles made from polystyrene (PS) and polyacrylic acid (PAA) were analyzed by SEM to measure the particle size [21]. Figure 5 shows the particles with size ranging from 1 to 7 μm. The above reported studies on characterization of polymeric membranes using SEM are summarized in Table 1.

#### 2.1.3. Limitations

In SEM, the sample should be in the solid form and free from moistures [22]. This is because SEM operates under vacuum and this will cause sample evaporation. Furthermore, the samples need to be electrically conductive and if not, coating is necessary. It should be noted that for pore-size measurement, SEM can be used for pores having a diameter in tens of micrometers. However, for gas separation membranes, SEM is not suitable for pore size measurements because the pore size is in the fractions of nanometer or units of Angstrom [23]. Furthermore, measurements of thin films of thickness less than 10 nm is not recommended due to the lower resolution of SEM [24]. In this case, high-resolution techniques such as transmission electron microscopy are required, which will be discussed in the following section.

**Table 1:** Performed studies using SEM for characterization of polymeric membranes.

Study	Membrane	Methodology	Conclusion	Ref.
Dense surface	Polyetherimide	Cut samples in liquid nitrogen. Gold coat samples.	No pores thus a dense membrane.	[15]
Pore size measurements	Polyethersulfone	Cut samples in liquid nitrogen. Used an imaging software to determine the pore size.	Average pore size of 30 nm.	[25]
Membrane thickness	poly(1-trimethylsilyl-1-propyne) (PTMSP)	Immersed samples in isopropanol. Cut samples in liquid nitrogen. Coated samples with gold.	Average PTMSP thickness of 1.5 μm.	[17]
Particle size measurements	Copolymer of polysulfone (PS) and polyacrylic acid (PAA)	Freeze-dried samples. Sputter-coated samples with platinum.	Particle size ranging from 1 to 7 μm.	[21]

### 2.2. Transmission Electron Microscopy

**Figure 6:**
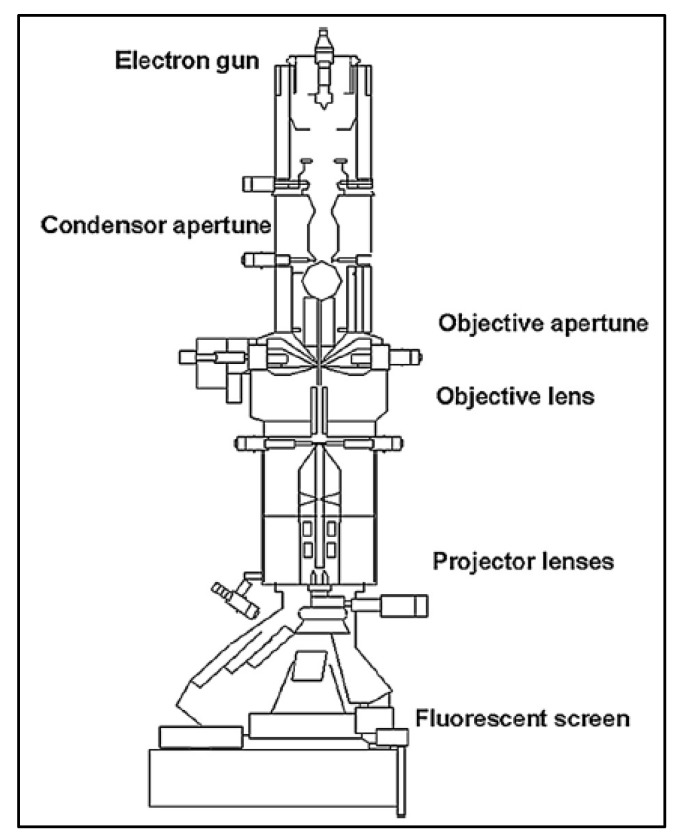
Components of transmission electron microscopy (TEM) instrument [32].

Transmission electron microscopy (TEM) is another technique for observing the membrane surface. Compared to SEM, the magnification in TEM can reach 50 million, making it more suitable for measurements in the nanometer scale [26]. In TEM, an electron gun is used, as a source of electrons, and pointed to the sample. The electrons pass through the sample and collected thereafter. These transmitted electrons will form an image in a fluorescent screen giving more details about the internal structure of the membrane [27]. The components of the TEM instrument are shown in Figure 6.

#### 2.2.1. Sample Preparation

Unlike SEM, TEM needs more preparation as the sample needs to be thin and small; usually less than 2.5 mm in diameter with thickness not exceeding 150 μm [27,28]. Before cutting the sample, most of the polymers are soft and it is suggested to harden them by immersing them in liquid nitrogen. To facilitate ion exchange and improve image contrast, the membranes are stained using a liquid containing positive stains that deposit electrons on the membrane surface such as sodium oxide and uranyl acetate [29]. After that, the sample is washed with deionized water and then dried. The sample is thereafter mounted on a TEM grid made of a metallic mesh and ready for analysis.

#### 2.2.2. Data Interpretation

**Figure 7:**
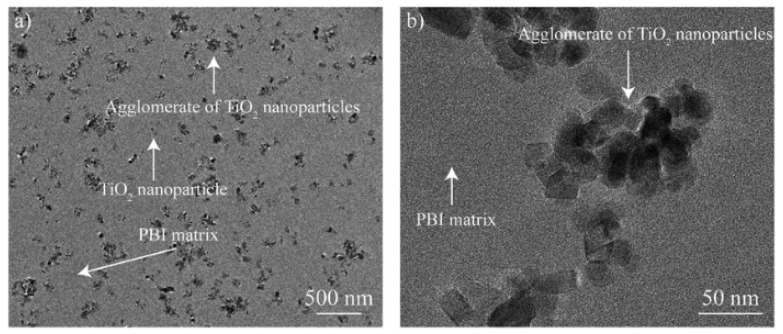
TEM images of TiO_2_ nanoparticles on polybenzimidazole (PBI) membrane with the addition of TiO_2_ nanoparticles: (**a**) At a magnification of 500 nm, (**b**) at a magnification of 50 nm [31].

TEM is widely used over SEM especially for analyzing mixed-matrix membranes made by adding nanoparticles to the polymer. The nanoparticles are expected to be uniformly distributed through the membrane [2]. However, agglomeration of the particles may occur due to molecular electrostatic forces [30]. TEM is an excellent instrument for studying the agglomeration of the particles. For example, Figure 7 shows the nanoparticles of titanium oxides deposited on polybenzimidazole (PBI) membrane for water vapor/gas separation. TEM revealed that some of the nanoparticles agglomerated but it is within the acceptable range [31].

**Figure 8:**
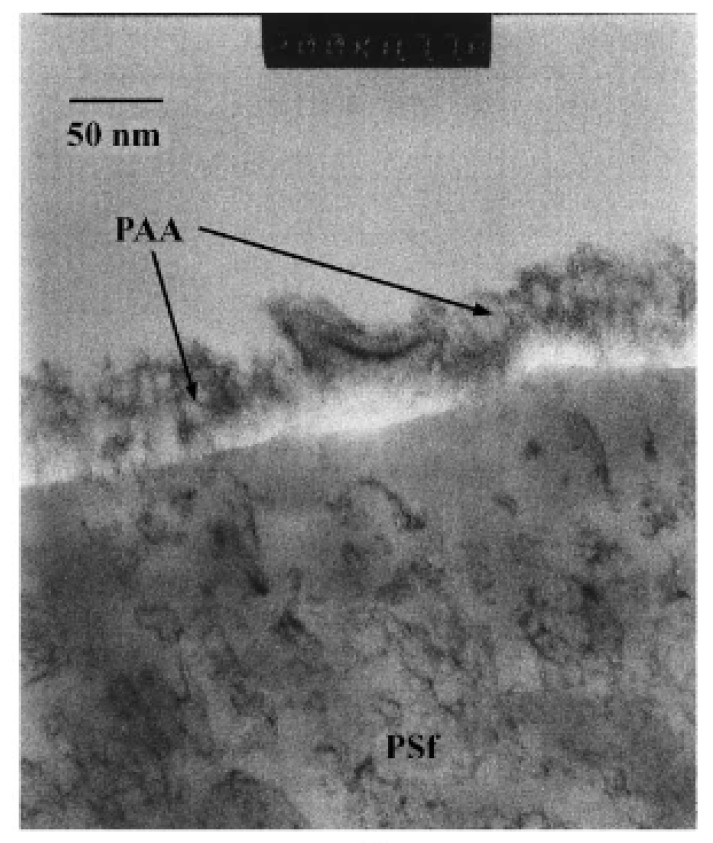
TEM image of a nano-film of grafted on polyacrylic acid (PAA) over polysulfone (PSf) [29].

Thin-film composite (TFC) membranes are made mainly by depositing a selective layer over a support. The film is usually in nanometers and TEM can be used to measure the film thickness. Figure 8 shows a film of polyacrylic acid (PAA) deposited on polysulfone (PSf). Based on TEM, the film thickness was about 20 nm [29]. The discussed characterization studies are summarized in Table 2.

#### 2.2.3. Limitations

Unlike SEM, TEM requires special preparation. The samples need to be generally no more than 2.5 mm in diameter. Also, the thickness should be no more than 100 μm, otherwise reduction in thickness will be required. Furthermore, TEM gives the image in 2D format and this does not show the topography data. Moreover, wet or volatile samples cannot be used in TEM due to the high vacuum, which causes sample evaporation and this may damage the instrument.

### 2.3. Atomic Force Microscopy

**Figure 9:**
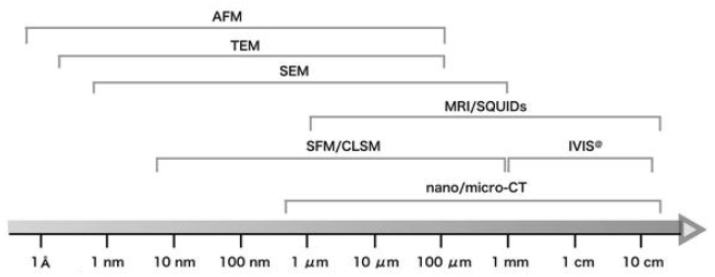
Differences between optical microscopy, SEM, TEM, and atomic force microscopy (AFM) in resolution [45].

Atomic force microscopy (AFM) is a high-resolution microscopy technique for surface examination. Figure 9 shows the scale difference between SEM, TEM, and AFM, and it is clear that AFM has a similar magnification power compared to TEM. Yet, AFM can provide 3D images of the surface that can provide surface topography along with surface roughness [33].

Unlike SEM and TEM, AFM works by measuring the force between a probe and the sample. The probe, which consists of a cantilever with a sharp tip, will try to touch the sample and this will cause the probe to deflect due to the attractive forces between the probe and the surface [34]. In reality, there are two modes for AFM operation: Contact mode and semi-contact mode. In contact mode, the probe will constantly touch the sample surface by adjusting the applied force, while in semi-contact mode, the probe will periodically touch the sample [35]. A laser beam will be used to detect the deflection and the reflected laser will pass through a photo-detector to form the image [36]. Because a probe is used, mechanical properties such as stiffness can also be measured by AFM based on the indentation [37].

#### 2.3.1. Sample Preparation

In AFM, liquid samples, in addition to solids, can be analyzed due to the absence of vacuum. Compared to SEM and TEM, AFM requires less or no preparation steps. For most polymers, coating with a conductive material or staining is not needed. It is advised however to cut the samples no larger than 10 by 10 mm with thickness up to 2 mm [38]. The sample is then fixed over a substrate made from a flat mica (silicate minerals) or a glass [39].

#### 2.3.2. Data Interpretation

**Figure 10:**
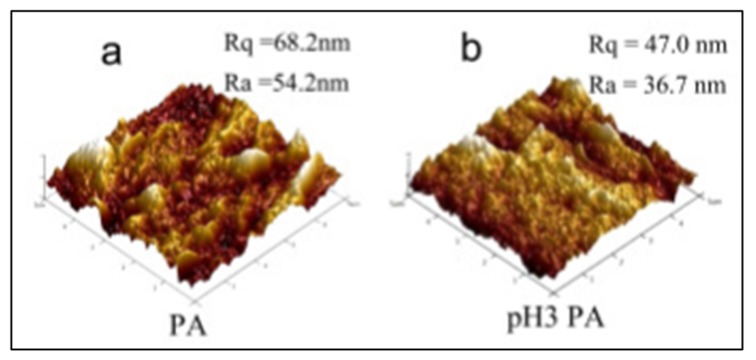
3D AFM data of polyvinylidene fluoride (PVDF)/polyvinylalcohol (PA) membrane: (**a**) Original surface, (**b**) surface after fouling due to the exposure to natural organic matter (pH3) [46].

AFM is capable of displaying the topography data of the membrane surface in the form of a 3D image. This information can be useful in studying the change in the membrane roughness due to fouling. With time, the rejected molecules will accumulate on the membrane surface causing a swelling, thus alternating the membrane roughness [40]. Figure 10 shows the topography data of polyvinylidene fluoride (PVDF)/polyvinylalcohol (PA) membrane before and after the operation. The data in Figure 9 are presented by two parameters, Ra and Rq. Ra mainly represents the roughness and it is the arithmetic average of the absolute values of the surface height deviations [41]. The higher the value of Ra, the rougher the surface. On the other hand, Rq represents the root mean square average of height deviation taken from the plane.

**Figure 11:**
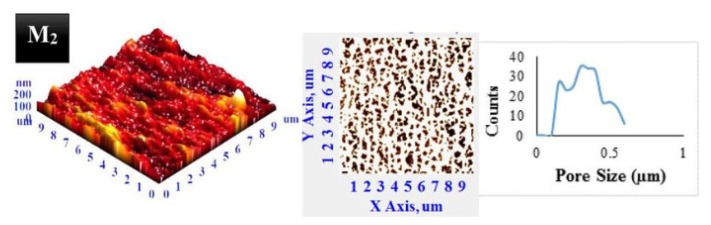
Determination of pore size distribution of polyvinylidene fluoride (PVDF) membrane by analyzing the 3D image generated by AFM [50].

AFM is also capable of determining the pore size distribution especially if the pore size of the membrane is larger than 2 nm [42]. Ultrafiltration membranes for water purification and waste-water treatments usually have pores with a size of 10 nm making AFM a suitable technique for pore size measurement [43]. Similar to SEM, pore size distribution can be determined by measuring the pore area of the generated images as shown in Figure 11 [44]. However, AFM provides better accuracy compared to SEM especially for mesoporous structure with pore size ranging from 2 to 50 nm.

**Figure 12:**
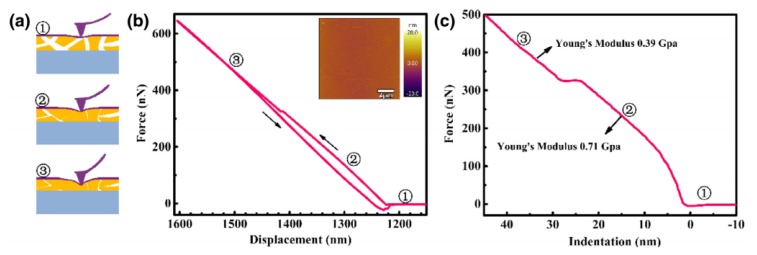
Use of AFM for the measurements of the stiffness of polymer of intrinsic microporosity (PIM-1) membrane by plotting the force versus displacement and indentation. Young’s modulus was calculated based on the Derjaguin–Muller–Toporov (DMT) method [51].

Because AFM works based on a moving probe, some mechanical properties can be measured by studying the indentation of the probe on the membrane surface. A graph of the force versus the displacement can be plotted and then converted to a plot of the stress versus the strain to calculate the stiffness of the membrane (Young’s modulus) as shown in Figure 12. In Figure 12b, the linear relationship between force and displacement indicates an elastic deformation. Young’s modulus values in Figure 12c were calculated using the Derjaguin–Muller–Toporov (DMT) method and the first value of 0.71 GPa at the surface showed an elastic deformation due to the elastic inner chains of the polymer. The change of Young’s modulus value to 0.39 GPa indicates less elasticity along with the membrane depth. It was also found later that the stiffer the membrane, the lower the gas permeability due to the reduction in free volumes [47]. These volumes provide the path for gas transport through the dense membrane. The discussed studies on using AFM for membrane characterization are given in Table 3.

#### 2.3.3. Limitations

AFM has some benefits over SEM and TEM in terms of sample preparation. However, AFM analysis requires more processing time. Also, AFM has a lower depth of field compared to SEM [48]. Nevertheless, the contrast in AFM images is generally better [49].

**Table 3:** Studies performed by AFM for membrane characterization.

Study	Membrane	Methodology	Conclusion	Ref.
Roughness measurements	Polyvinylidene fluoride (PVDF) /polyvinylalcohol (PA)	Samples in contact mode with silicon nitride probe. Scan area of 5 μm by 5 μm. Five areas were analyzed.	Roughness decreased from 54 to 37 nm indicating a fouling.	[46]
Pore size distribution	Polyvinylidene fluoride (PVDF)	Dried samples at 40 °C overnight. Semi-contact mode. Image processing by NT-MDT software.	Average pore size of 0.3 μm.	[50]
Membrane stiffness	Polymer of intrinsic microporosity (PIM-1)	Contact-mode analysis. Probe radius of 8 nm. Derjaguin–Muller–Toporov (DMT) method to calculate Young’s modulus.	Elasticity decrease with membrane depth.	[51]

## 3. Crystal Structure Analysis

### 3.1. X-ray Diffraction

X-ray diffraction (XRD) is a technique for studying the crystal structure of the membrane. The technology is unique in determining the crystal structure type and the distance between the polymeric chains [52]. Moreover, the polymer structure, either glassy or amorphous, can be identified by XRD. Glassy polymers feature ordered, more crystallized structure with better mechanical properties [53,54,55]. They are widely used for gas separation due to the high selectivity [56]. This means that they separate gases with a higher product purity. The high selectivity in glassy polymers is related to the high gas solubility due to the excess of free volumes [57].

On the other hand, amorphous (rubbery) polymers have a random structure but with lower mechanical properties [58]. However, high gas permeability is achieved by amorphous membranes due to the absence of gas diffusion limitation [59]. In addition to crystal structure studies, the chemical compounds presented in the sample can be identified by XRD. This information is valuable for confirming sample purity. The formation of secondary phases due to membrane degradation can also be spotted by XRD.

**Figure 13:**
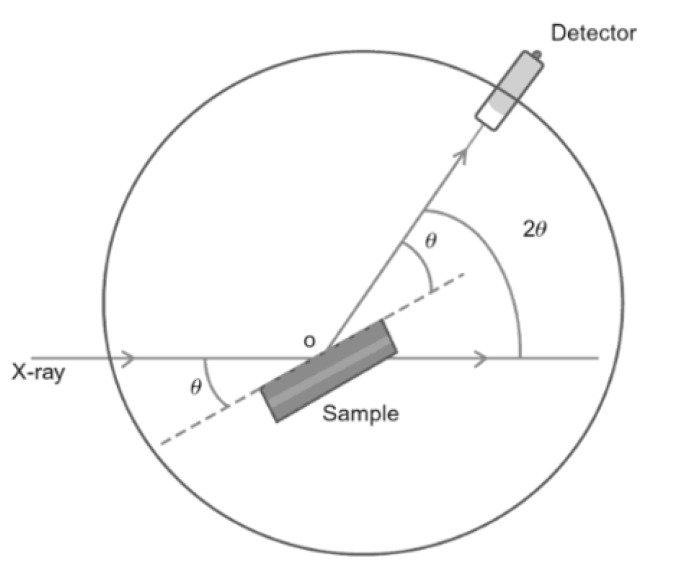
XRD instrumentation diagram [62].

**Figure 14:**
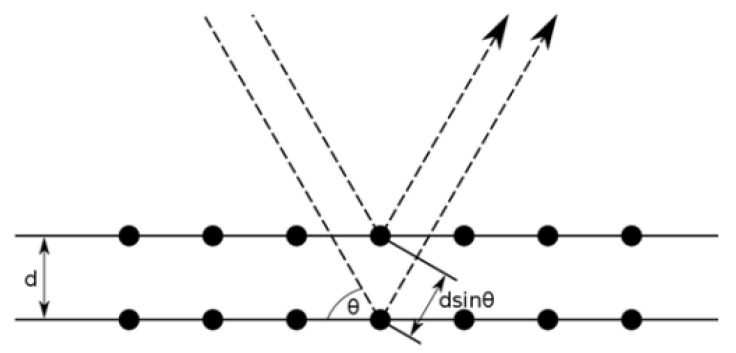
Diffraction of an X-ray beam from a sample [63].

In XRD, a filament such as tungsten is used to generate an X-ray beam [60]. This beam is directed to the sample and the scattered X-ray is detected and analyzed. Figure 13 shows how XRD analysis is performed. Some X-rays will be reflected upon hitting the surface while some will enter the surface and then reflect as shown in Figure 14. This variation is called diffraction and Bragg’s law can be applied to calculate the diffraction angle (2θ) [61]:

(1) nλ=2dsinθ

where n is the order of reflection (usually unity), λ is the wavelength and it is determined from the source of radiation used (commonly copper K-α), and *d* represents the plane distance between a set of atoms.

#### 3.1.1. Sample Preparation

XRD is a non-destructive test with minimum preparation. It can be used to analyze solids, as well as powders. For solid membranes, the samples are usually cut into small sections of 20 by 20 mm. The sample is then mounted on the XRD chamber. For polymer powders, generally, 100 to 200 mg of powder is needed and spread over a carbon tape. This tape has a very low XRD intensity and will not affect the analysis [64].

#### 3.1.2. Data Interpretation

The data in XRD are represented by intensity (counts) versus diffraction peak angle (2θ). Usually, the angle starts from 10 to 80 and each compound has a defined set of peaks. For example, polyetherimide is known to have sharp peaks at 21.4 and 23.8 (2θ) [15]. If additional peaks were detected for a pure polyetherimide membrane, this may indicate impurities or membrane degradation.

**Figure 15:**
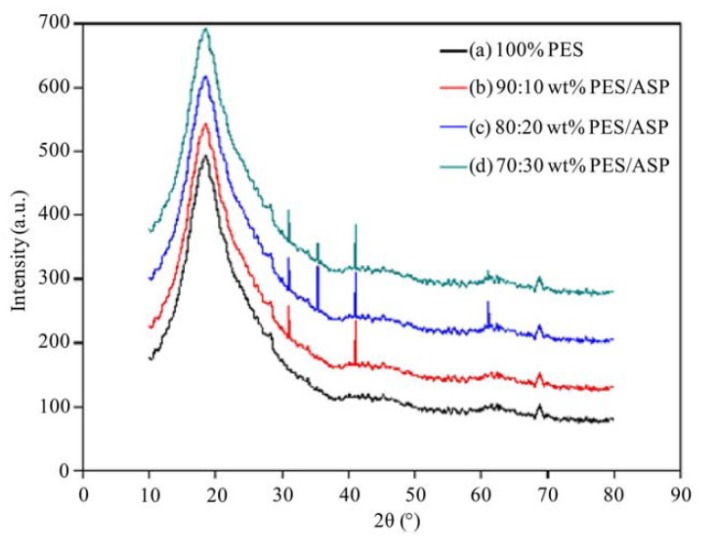
XRD data of a mixed-matrix membrane of polyethersulfone membrane with aluminosilicate particles [66].

In polymeric membranes, there is a tradeoff between permeability and selectivity due to the nature of the polymer [1]. Mixed-matrix membranes can overcome this limitation by adding fillers of another material to the polymer during the membrane preparation [65]. XRD can be beneficial for studying the effect of adding the fillers to the polymer matrix. The fillers are supposed to be detected in the XRD peak profile along with the polymer. For example, a mixed-matrix membrane was made by adding aluminosilicate particles to polyethersulfone with different concentrations [66]. XRD data revealed that the intensity of polyethersulfone peaks increased making the structure more crystallized, which enhanced the mechanical properties (Figure 15). Some researchers also observed the relationship between the intensity and gas permeation and they concluded that the higher the intensity, the lower the permeation [67]. This was interpreted by the limitation in gas diffusion through the membrane as the structure becomes more crystallized [68].

**Figure 16:**
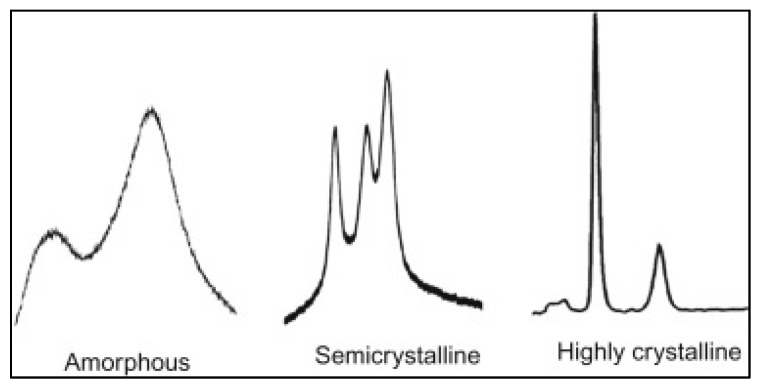
Differentiation of XRD peaks for determining the glassy and amorphous structures [62].

Amorphous and glassy structures can be distinguished in XRD by observing the peak shape [62]. Sharp peaks refer to a more crystallized structure while broad peaks refer to an amorphous structure as demonstrated in Figure 16. *d*-space (*d*) is another important parameter for membrane characterization. In Equation (1), *d* represents the distance between two planes of atoms. In polymeric membranes, *d*-space can represent the distance between the polymer chains and it is calculated based on the highest peak of the XRD pattern. Long-chain polymers are presumed to have a higher *d*-space value. In polymer science, it is observed that the longer the chain, the less the structure crystallinity due to the transformation from a glassy to a rubbery structure [52]. In terms of gas permeability, it is easier for the gas to pass through an amorphous structure due to the absence of diffusion limitation. This was also confirmed by noticing an increase in the membrane permeability for longer chain polymers [69]. Table 4 summarizes the mentioned studies for membrane analysis by XRD.

#### 3.1.3. Limitations

X-ray beam does not interact strongly with lighter elements and this may limit the elements detection in XRD [70]. Also, XRD is more accurate for measuring large crystal structures rather than small ones [71]. Furthermore, if more compounds are presented in the sample, peak overlap may complicate the analysis.

### 3.2. X-ray Scattering

**Figure 17:**
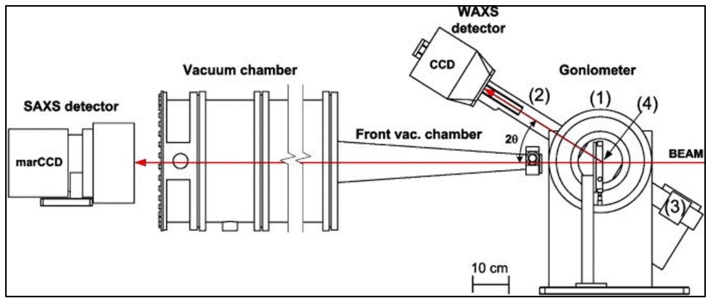
Small-angle X-ray scattering (SAXS)/wide-angle X-ray scattering (WAXS) instrument for crystal and molecular analysis: (1) Rotation unit, (2) arm, (3) counterweight, and (4) hot stage [79].

XRD is performed widely for materials with well-ordered (crystalline) structure. However, for semi-crystalline or non-crystalline (amorphous) materials, X-ray scattering is preferred [72]. In X-ray scattering, a monochromatic beam of X-rays is sent to the sample in which some of the X-rays will be scattered. These scattered X-rays are then detected and analyzed. There are two types of X-ray scattering: Small-angle X-ray scattering (SAXS) and wide-angle X-ray scattering (WAXS). In SAXS, the scattering angle (2θ) is less than 5° and the technique focuses on studying the particles in a system. For instance, SAXS can determine the nanoparticle size distribution and pore size [73,74]. These properties can still be measured by microscopic techniques, however SAXS is better in giving the average values for larger areas [75]. On the other hand, WAXS scans at a scattering angle of over 5° with the ability to study the degree of crystallinity and the chemical composition [76]. The SAXS and WAXS are usually combined in one instrument and its components are given in Figure 17.

#### 3.2.1. Sample Preparation

SAXS and WAXS barely need sample preparation for polymeric membranes similar to XRD.

#### 3.2.2. Data Interpretation

**Figure 18:**
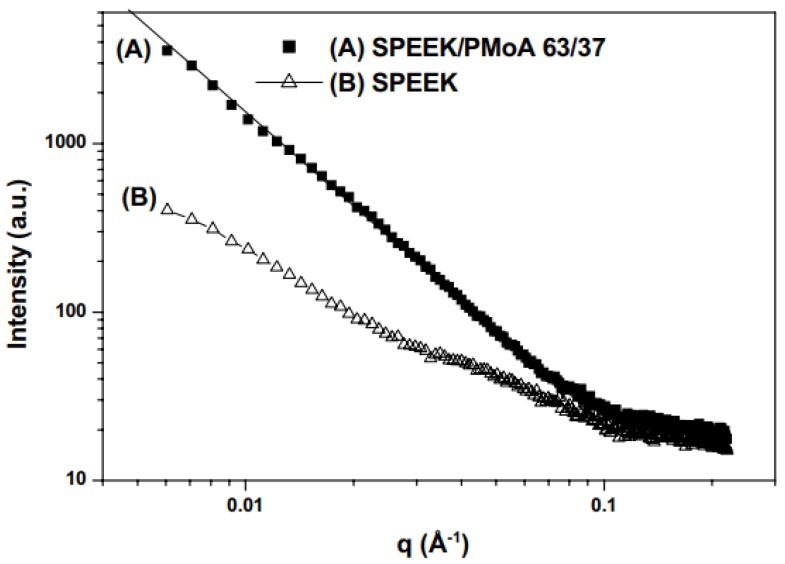
SAXS analysis of sulfonated poly(aryl ether ketone) (SPEEK)/phosphomolybdic acid (PMoA) membranes (**a**), and pristine SPEEK membranes (**b**) [80].

**Figure 19:**
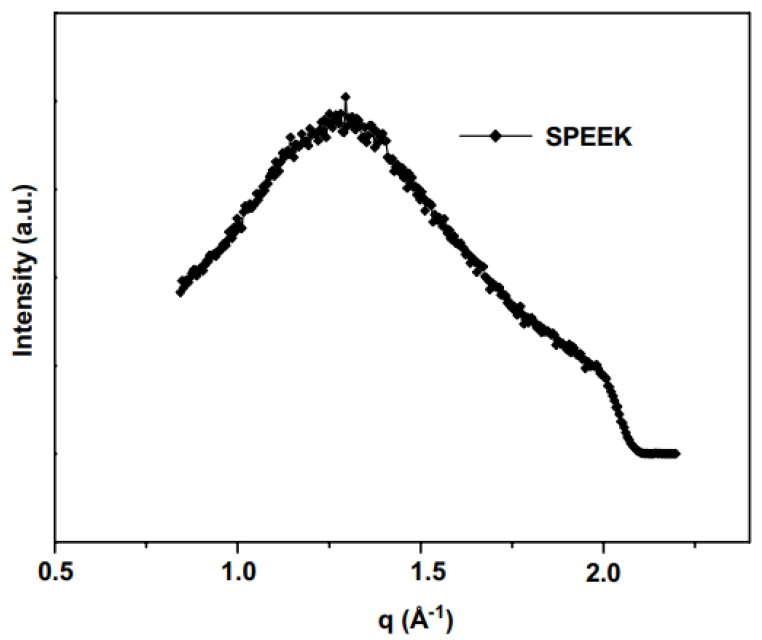
WAXS analysis revealed an amorphous structure of SPEEK due to the broad peak [80].

The data in SAXS/WAXS are presented by the intensity and the scattering vector (*q*) and the latter is calculated by:

(2) $$q=\frac{4\pi }{\lambda }sin\left(\frac{\theta }{2}\right)$$

where *λ* is the wavelength of the incident X-rays and θ is the scattering angle. SAXS was used to study the preparation method of sulfonated poly(aryl ether ketone) (SPEEK) membranes. The absence of the ionomer peak (–SO_3_H) indicated no nano-phase separation between the hydrophilic segments and the polymer matrix (Figure 18). Addition of inorganic filler resulted in a significant increase in intensity. The high intensity revealed that the filler was merged into the mass-fractal structure meaning that PMoA was successfully crosslinked with the polymer [77]. SAXS was also used to calculate the particle radius (*L*_m_) in the solution using Schimdt equation:

(3) $$L_{m}=\frac{\pi }{q_{\min}}$$

where $q_{\min}$ is the lower limit of the scattering vector (*q*) in SAXS graph. PMoA particles were found to have an average radius of 524Å. In addition to the above, WAXS was implemented to calculate the pore size distribution of PIM membranes. Equation (3) was used to calculate the pore size but with the lower and upper limit of scattering vector (Figure 19). The intensity is proportional to the amounts of pores having that pore size. The reported pore size for amidoxime-grafted PIM membranes ranged from 3.9 to 5.9 Å [78]. Table 5 summarizes the studies for membrane characterization by SAXS and WAXS.

#### 3.2.3. Limitations

Sample homogeneity is critical for SAXS analysis as sample aggregation or degradation will result in incorrect data [81]. Sample damage by radiation is also possible and can distort the data [82]. Furthermore, the scattered intensity can be weak for some systems [83].

## 4. Functional Groups Analysis

### 4.1. Fourier-Transform Infrared Spectroscopy

Fourier-transform infrared spectroscopy (FTIR) is a technique to determine the functional groups. Usually, the functional groups give the chemical reaction properties of the compound. Some examples of the functional groups are hydroxyl (OH–) and carbonyl (C=O). If the functional groups are known, the compound class can be determined. For example, the hydroxyl group (OH–) usually represents alcohols and carboxyl acids and FTIR can easily distinguish between those compounds as discussed later.

**Figure 20:**
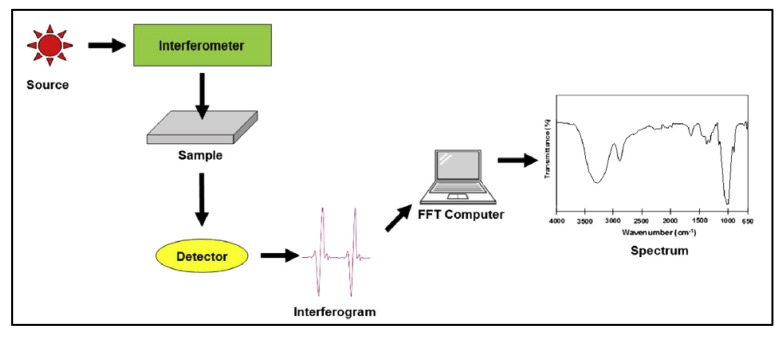
Components of FTIR spectroscopy for detection of functional groups [32].

In FTIR spectroscopy, the analysis can be performed on gases, liquids, and solids. It is a quantitative technique as well [84]. It works based on sending infrared radiation to the sample with the use of a reference solvent [85]. Some of the radiation will be absorbed by the sample and others will pass through or will be transmitted. A mathematical model (Fourier transform function) will be used to covert the data into the spectrum. Figure 20 shows the components of the FTIR spectrometer.

Attenuated total reflection (ATR) is another method for generating FTIR spectrum without the use of a reference material. In ATR, a beam of infrared light is sent to the sample through ATR crystal made mainly from diamond [86]. This will cause creation of evanescent waves. The reflected beam is then detected and analyzed to generate the FTIR data.

#### 4.1.1. Sample Preparation

FTIR spectroscopy usually requires sample preparation before analysis. Generally, the membrane will be crushed into a powder of 2 to 5 mg then mixed with a solvent commonly potassium bromide with a mixing ratio of 1 to 100 [87,88]. Then, the mixed powder is pressed in a die at a load of 10 tons to form a pellet of 13 mm [89]. The pellet is then inserted in the FTIR chamber for analysis. It should be mentioned that potassium bromide is usually used as a reference material because it does not interfere in the infrared window from 400 to 4000 cm^−1^ [90]. Also, it acts as a carrier for the infrared spectrum. Furthermore, the reference material of potassium bromide will be used to plot the transmittance axis in the FTIR graph. Modern FTIR techniques such as ATR requires little or no sample preparation with the ability to analyze solid samples [91].

#### 4.1.2. Data Interpretation

The data in FITR spectroscopy is given by the transmittance versus the wavelength. Zero transmittance means that the sample absorbed all the radiation while 100% transmittance means that the sample absorbed the same amounts of radiation as the reference. The generated graph is unique for each molecule and this can be used in identifying the functional groups. For instance, the hydroxyl group (OH–) represents both alcohols and carboxylic acids but FTIR can differentiate between them based on the wavelength. For instance, alcohol’s peak is detected at a wavelength of 3200 to 3600 cm^−1^ while carboxylic acids peak is detected at 2500 to 3300 cm^−1^ [92]. Table 6 shows the wavelength of different functional groups for FTIR analysis.

**Figure 21:**
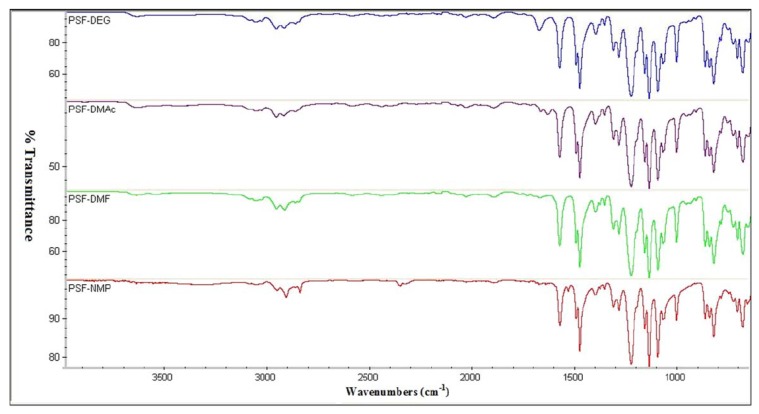
FTIR analysis indicating no significant change in the preparation of polysulfone (PSF) membrane using diethylene glycol (DEG), dimethylacetamide (DMAc), dimethylformamide (DMF), and n-methylpyrrolidone (NMP) as solvents [93].

FTIR spectroscopy can be very useful for determining the compatibility between the polymer and the solvent. The membrane preparation involves the use of solvent for casting. The solvent should not react with the polymer to avoid changing the physical and chemical properties of the polymer [93]. A study was made by preparing a PSf membrane with different solvents such as diethylene glycol (DEG), dimethylacetamide (DMAc), dimethylformamide (DMF), and n-methylpyrrolidone (NMP). FTIR data is given in Figure 21 and none of the solvents altered the FTIR spectrum indicating a pure PSf membrane. In mixed matrix membranes, FTIR analysis is widely used to determine the interaction between the polymer and the fillers for better compatibility [94]. This can be studied by detecting the functional group of the fillers within the FTIR spectrum of the mixed matrix membrane. Table 7 sum up the discussed membrane characterization studies by FTIR.

#### 4.1.3. Limitations

As discussed before, FTIR may require sample preparation but for modern FTIR techniques, almost no preparation is needed. Aqueous solutions are difficult to analyze by FTIR due to the strong infrared absorption of water [95]. Furthermore, FTIR cannot detect molecules of two identical atoms such as oxygen and nitrogen as they do not absorb infrared radiation [96].

**Table 6:** Identification of chemicals based on the wavelength of the functional groups by FTIR spectrometer [97].

Wavelength (cm^−1^)	Functional Group	Chemical Class
3200–3500	OH–	Alcohols
2500–3300	OH–	Carboxylic acids
2800–3000	N–H	Amine salts
3267–3333	C–H	alkynes
3000–3100	C–H	alkenes
2840–3000	C–H	Alkanes
2349	O=C=O	carbon dioxide
1380–1415	S=O	sulfates

**Table 7:** FTIR studies for characterization of polymeric membrane.

Study	Membrane	Methodology	Conclusion	Ref.
Polymer-solvent compatibility	Polysulfone	Not reported.	Functional groups of only aryl ethers, aryl sulfones and methyl were detected indicating no interaction with the solvent (solvent is compatible).	[93]
Polymer-filler interaction	Polyetherimide with metal-organic framework filler (MIL-53)	FTIR Spectrum recorded at 4000–500 cm^−1^.	Peaks of (C-N), (Si-O), (CO_2_–), and (Al-O) indicated that MIL-53 was successfully incorporated in the polymer matrix.	[94]

### 4.2. Raman Spectroscopy

**Figure 22:**
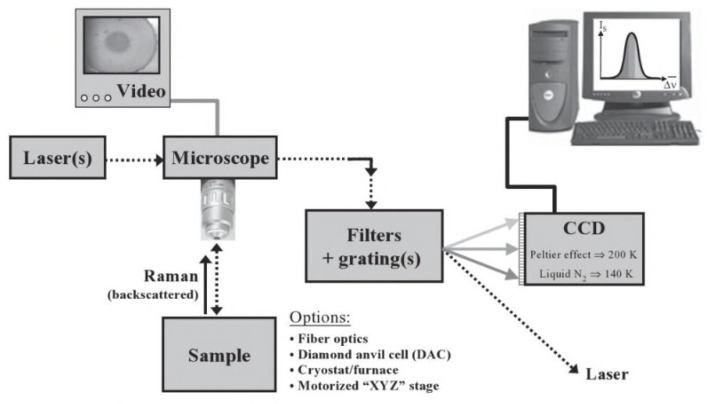
Components of Raman spectroscopy instrumentation [103].

Raman spectroscopy studies the vibration and rotation modes of molecules [98]. Each compound has its own unique spectrum that can be cross-referenced with a set of known Raman spectrum. Basically, laser light is focused on the sample and this light will cause molecular vibration. This form of excitation will cause light scattering that results in shifting up or down the laser photon energy [99]. Components of Raman instrument are given in Figure 22.

Raman spectroscopy is a quantitative and qualitative technique that gives information about the functional groups in the polymer. Compared to FTIR, Raman is more sensitive to the functional groups giving sharper peaks [100]. Furthermore, diatomic molecules such as oxygen and nitrogen can be detected in Raman spectroscopy. Also, Raman spectrometer is an excellent tool for studying the changes in the polymer crystal structure due to changes in the chemical and mechanical properties [101]. Furthermore, polymer chain orientation and interfacial surface properties can be obtained by Raman spectrum [32].

#### 4.2.1. Sample Preparation

Raman is considered as a non-destructive test and the preparation is much easier compared to other methods such as FTIR where dissolving the sample with a proper solvent may be needed. The sample area for Raman spectrometer is usually less than 10 cm^2^ with thickness less than 2.5 cm placed over a glass or silicon substrate [102].

#### 4.2.2. Data Interpretation

**Figure 23:**
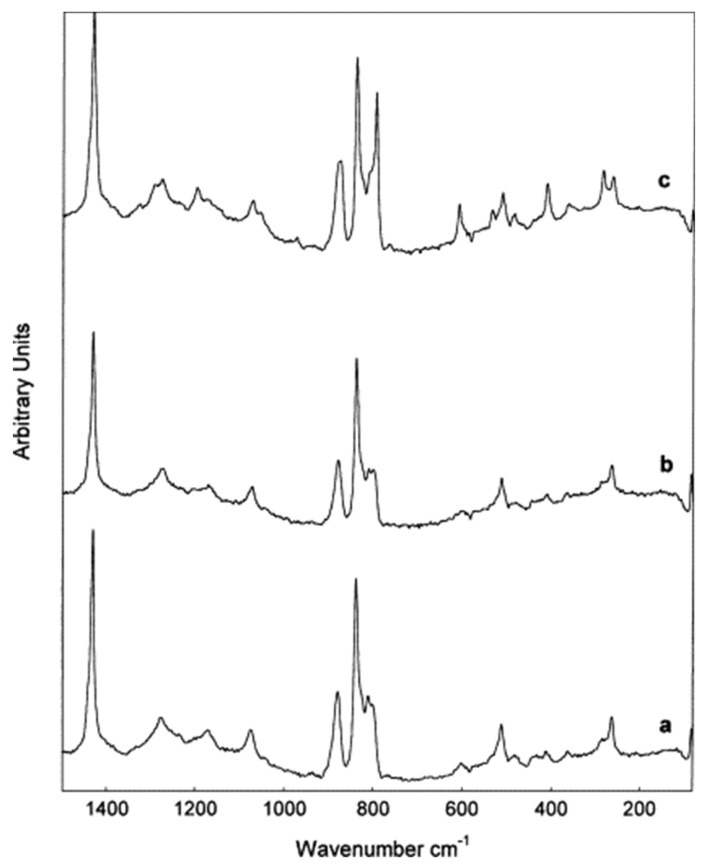
Raman spectroscopy of PVDF membrane using different solvents: (**a**) DMF; (**b**) NMP; (**c**) triethyl phosphate (TEP) [101].

The data in Raman spectroscopy is given in the form of intensity over wavelength of the scattered photons (Raman shift). The technique can be used to determine the form of the crystal structure by observing the intensity peaks. For example, the change in intensity of the same peak indicates a variation in the structure asymmetry. For instance, PVDF membrane is known to have three different structures; form I (all trans structure), form II (trans-Gauche sequence structure), and form III (three trans bonds separated by Gauche structure) [101]. Raman spectroscopy distinguished between these structures by noticing the peak shape and intensity at a wavelength of 793 cm^−1^. The use of various solvents such as DMF, NMP, and triethyl phosphate (TEP) for PVDF membrane preparation resulted in different structures as shown in Figure 23. Form II structure of PVDF was recognized by the additional peak at 795 cm^−1^ (Figure 23c).

**Figure 24:**
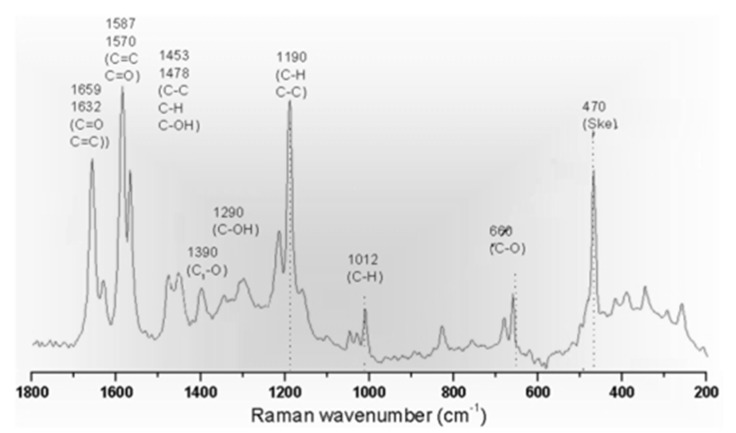
Functional groups identified by Raman spectroscopy and the corresponding wavelength [110].

Raman peaks can also be used to determine the functional groups of the polymer. For example, ethylene compounds with functional group of C=C can be recognized at a wavelength of 1623 cm^−1^. Some chemicals have the same functional group such as ketones and aldehydes in which they share the carbonyl (C=O) group. Raman spectroscopy can differentiate between ketones (RCOR’) and aldehydes (RCHO) at the wavelength of 1659 and 1725 cm^−1^, respectively [104,105]. Figure 24 demonstrates the peaks and wavelengths of different functional groups.

In addition to the above, Raman spectroscopy can be utilized to study the aging effect of the membrane due to operation. For example, the presence of new functional groups within Raman spectrum may indicate degradation. Another way to detect membrane degradation is by observing the transformation of the main backbone into shorter chains [106].

Raman spectroscopy is a quantitative technique as well and for some mixtures, the intensity can be related to the amounts of the compound in the sample. To illustrate, the volume of methanol in a sample increased linearly with the decrease of intensity peak at 729 cm^−1^ and this was used for the quantification [107]. The mentioned studies by Raman spectroscopy for membrane characterization are given in Table 8.

#### 4.2.3. Limitation

Unlike the FTIR spectrometer, Raman spectroscopy requires a longer time for the analysis. Due to the use of laser in Raman spectroscopy, some samples may release fluorescent light that interferes with the spectrum causing an increase in the background noise [108]. Moreover, polar molecules often have weak Raman signals as the atoms hold electrons so closely [109].

**Table 8:** Raman studies for characterization of polymeric membrane.

Study	Membrane	Methodology	Conclusion	Ref.
Crystal structure	Polyvinylidene fluoride (PVDF)	Membrane layers were superimposed in a solid sampler. Spectrum recorded at 2 cm^−1^ resolution.	Additional peak at 795 cm^−1^ indicated formation of trans-Gauche sequence structure.	[101]
Membrane degradation	Perfluorinated sulfonic-acid (PFSA)	Samples mounted vertically. He-Ne laser and Peltier-cooled charge coupled device (CCD) to detect Raman spectrum. Resolution of < 2 cm^−1^.	Decrease in intensity of C–O–C, C–S and S–O bonds indicated membrane degradation.	[111]

### 4.3. Nuclear Magnetic Resonance Spectroscopy

**Figure 25:**
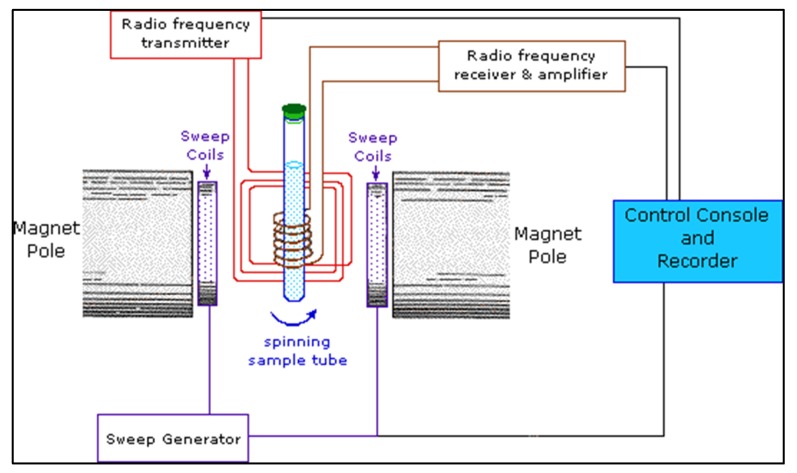
Components of NMR instrument [122].

Nuclear magnetic resonance (NMR) spectroscopy is another technique for analyzing the molecular structure and identifying the functional groups. It is also used to study polymer blend miscibility and polymer degradation [112,113]. NMR spectroscopy is based on placing the sample between two poles of a powerful magnet [114]. The sample will spin and radio waves will be broadcast to the sample. This will cause excitation of the nuclei and this will generate a resonance frequency detected by a radio receiver as shown in Figure 25. Compared to Raman, NMR spectrum has less background interference and it is capable of detecting polar compounds [115].

#### 4.3.1. Sample Preparation

Similar to FTIR spectroscopy, the sample in NMR spectroscopy needs to be in the liquid form. Generally, the membrane is cut into small pieces having a mass of 20 to 50 mg [116]. The sample is then dissolved in a strong solvent such as deuterated chloroform [117]. The concentration of the sample in the mixture should be about 1 wt% [118]. The liquid is then placed in a 5 mm tube and ready for analysis [119].

#### 4.3.2. Data Interpretation

The data in NMR spectroscopy is presented by the chemical shift (δ) in parts per million (ppm). This property is calculated from the resonance frequency of the sample (*v*) with a reference:

(4) $$\delta =\frac{v_{\mathrm{sample}}-v_{\mathrm{ref}}}{v_{\mathrm{ref}}}$$

**Figure 26:**
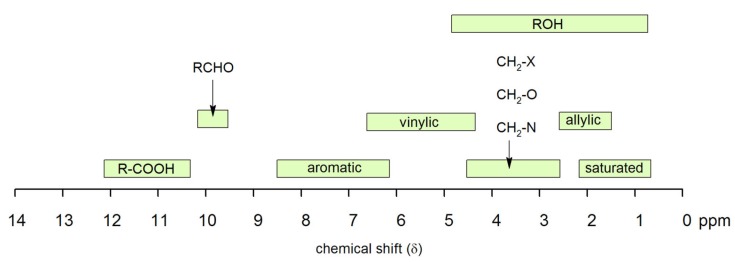
NMR chemical shift for various classes and functional groups [127].

Routinely, tetramethylsilane (TMS) is used as a reference and it has a chemical formula of Si(CH_3_)_4_. TMS has a sharp resonance line with its 12 protons [120]. Furthermore, TMS is inert with most samples. The chemical shift is unique for each functional group as given in Figure 26.

**Figure 27:**
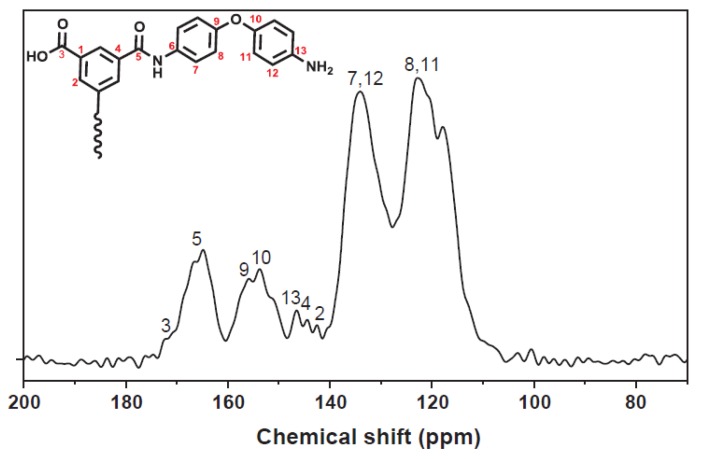
Use of NMR for study the molecular structure of polyetherimide thin film composite membrane [19].

NMR spectroscopy was used to determine the functional groups of a polyetherimide thin-film composite membrane made from 4,4′-oxydianiline (ODA) and trimesoyl chloride [19]. The technique was useful in determining the molecular structure of the composite membrane by detecting compounds such as carboxylic acid at 172 ppm, phenyl rings attached with oxygen at 155 ppm, and an aromatic ring attached to amine at 146.5 ppm (Figure 27).

**Figure 28:**
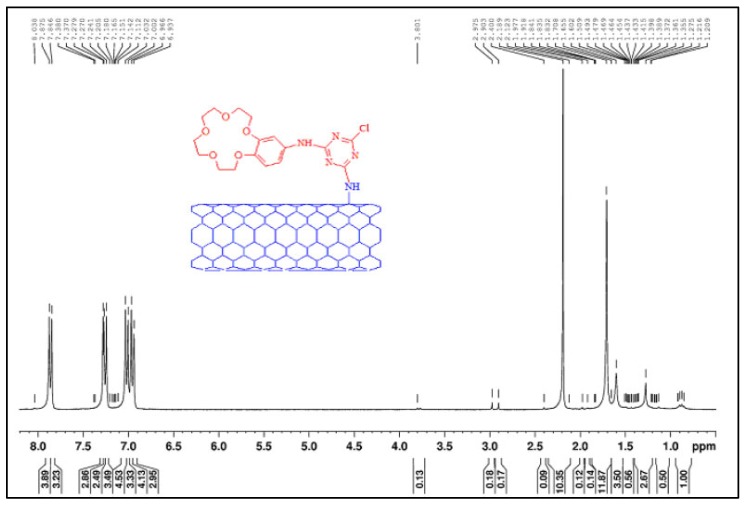
NMR analysis of a mixed matrix membrane made from polysulfone (PSf) and carbon nanotubes showing the detection of amino-benzo-crown-ether group at 2.975 ppm [121].

Functionalization refers to adding a chemical group into the polymer molecules. It can enhance the polymer physical, mechanical, and chemical properties. For instance, amino-functionalized carbon nanotubes were added to polysulfone (PSf) during the membrane preparation to improve the polymer reactivity [121]. It is known that the functionalization reaction of amino-carbon nanotubes with the polymer will result in formation of new functional groups. NMR was used to analyze the mixed-matrix membrane and functional group such as NH_2_ protons was detected at 1.845 ppm. The amino-benzo-crown-ether group was measured at 2.975 ppm, which was attributed to the –NH– bond confirming the functionalization reaction (Figure 28).

In addition to studying the chemical structure, NMR can be used to study the polymer blend miscibility. Two or more polymers can be blended to form a single membrane. NMP can be implemented to study the interaction between the polymers by observing the changes in the intensity in chemical shift. For instance, the miscibility between polysulfone and polyvinyl methyl ether (PVME) were studied at a weight percentage of 65 wt% and 35 wt%, respectively. NMR was performed for pure polysulfone and PVME for comparison. The significant increase in polysulfone intensity in the polymer mixture indicated a good miscibility [123].

NMR spectroscopy is an excellent tool for monitoring the membrane degradation. With time, the membrane performance reduces due to accumulation of the rejected molecules or due to the reaction with the feed impurities [124]. It is known that some acidic environments can accelerate membrane degradation and this can be studied by detecting the free radical peaks by NMR [125]. Furthermore, the disappearance of some chemical shift peaks indicates membrane degradation [126]. For instance, a perfluorinated ionomer membrane (Nafion^®^ 117) has been examined for degradation due to fuel cell operation. It was found that the C–F bond was decomposed to fluoride and sulfate ions indicating a membrane decomposition. Moreover, detection of hydroxyl and hydroperoxyl radicals also confirmed Nafion^®^ degradation [125]. Table 9 summarizes the mentioned studies in this paper for membrane analysis by NMR.

#### 4.3.3. Limitations

Paramagnetic elements such as oxygen and sodium have less NMR signals [128]. This is because those elements lose their magnetic properties after the magnet is removed. Furthermore, the quantitative analysis of heavy elements may take hours to complete [115].

## 5. Elemental Composition Analysis

### 5.1. Energy-Dispersion X-Ray Spectroscopy

**Figure 29:**
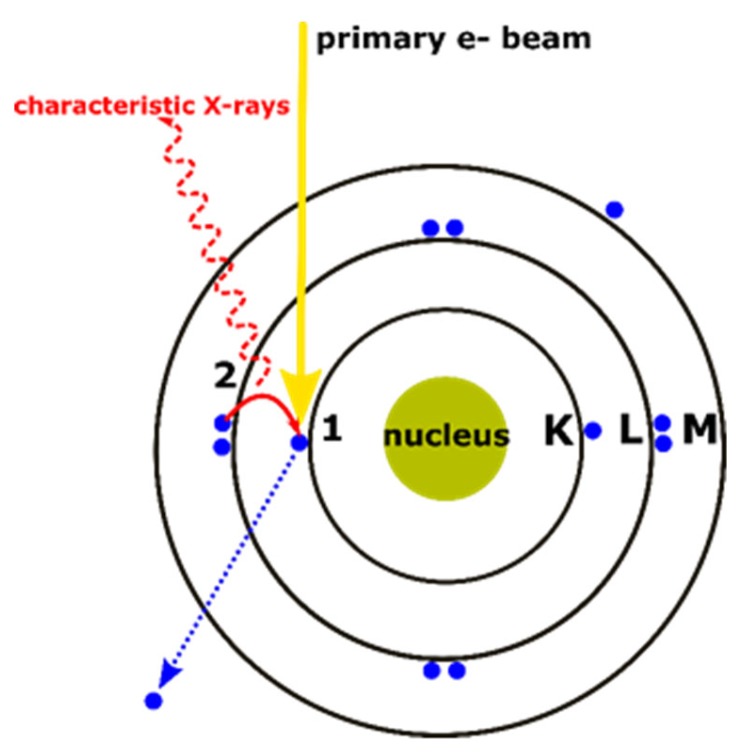
Atom excitation by electron and the release of -rays for EDS analysis [133].

Energy-dispersion X-ray spectroscopy (EDS) is a method for detecting the elements of a sample. EDS also gives the mass fraction of each element. EDS is considered as a “bulk” analysis technique that covers a larger area with a higher depth of generally of 1 μm [129]. The analysis processing time is also fast and data are displayed within seconds. EDS is commonly integrated with the SEM because both EDS and SEM share the same electron beam. This beam will excite the atom and will cause the release of electrons. The electrons from a higher state will move to fill in the vacancies and this will emit X-rays to balance the energy difference between the electrons (Figure 29). When the X-rays hit the EDS detector, pulses are created and then converted to a voltage by an analyzer in which the voltage is proportional to the energy of the X-rays [130].

#### 5.1.1. Sample Preparation

The prepared samples for SEM can be directly analyzed by EDS. However, if the sample was coated due to poor conductivity, the coating material will be detected in the EDS spectrum. Therefore, the coating material should be carefully selected so it cannot overlap with the elements found in the sample.

#### 5.1.2. Data Interpretation

Data of EDS analysis are presented by the intensity in counts and X-ray energy in keV. Each element has a peak at specific X-ray energy and this information can be utilized as a reference to pinpoint the element. EDS is a quantitative technique too and the composition can be calculated based on the intensity. This is useful as well for determining the chemical formula of the sample. Actually, EDS gives the data in atomic composition then the data are used to calculate the chemical formula. For example, to calculate the formula of a compound containing two elements (A and B), the following equation can be used:

(5) $$A_{x}B_{y}$$

where *x* and *y* are the atomic percentages determined by EDS. Now, the formula should be normalized by the lowest number (assuming x is the lower number):

(6) $$AB_{y/x}$$

**Figure 30:**
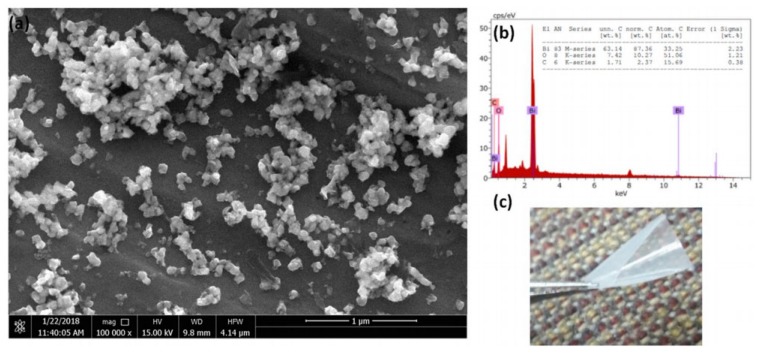
(**a**) SEM image of Bi_2_O_3_ particles deposited on poly(vinyl alcohol) membrane, (**b**) EDS analysis of Bi_2_O_3_, (**c**) membrane image [132].

The mass percentage can be then calculated using the atomic weight and the atomic number of the elements:

(7) $$x\left(wt\%\right)=\frac{x\left(at\%\right)\times m_{x}}{x\left(at\%\right)\times m_{x}+y\left(at\%\right)\times m_{y}}$$

where *m_x_* and *m_y_* are the atomic masses of element *x* and *y*, respectively. EDS can also be used to monitor any changes in the membrane composition due to operation. For example, the sulfur content in polypiperazine-amide membrane was measured to study the membrane fouling due to coal mine drainage [131]. EDS assisted the chemical cleaning method to restore the membrane by measuring the sulfur after the treatment. Furthermore, EDS was used to determine the chemical formula of nanoparticles inserted in a poly(vinyl alcohol) membrane [132]. EDS matched the added filler of bismuth(III) oxide with the formula of Bi_2_O_3_ as given in Figure 30. EDS studies for characterization of polymeric membranes are given in Table 10.

#### 5.1.3. Limitations

Unfortunately, EDS cannot give the full analysis of polymers due to the inability to detect hydrogen. This is because hydrogen has no electrons in the k-shell [134]. However, EDS can still be useful to detect elements that are usually presented in polymers such as carbon, nitrogen, and oxygen. Nevertheless, the quantitative analysis of EDS is more effective for elements with high atomic mass starting from 20 and above [135]. Because polymers are organic materials with mainly hydrogen, carbon, nitrogen, and oxygen, it is not suggested to use EDS to quantify the polymer. This is because the low atomic mass elements produce weak X-ray signals and therefore the composition data might be inaccurate. The typical detection limit of EDS is 0.1 wt% or 1000 ppm. This means that EDS cannot detect elements below that range. The general error expected from EDS is between -2 to 2 wt%.

### 5.2. X-Ray Fluorescence

**Figure 31:**
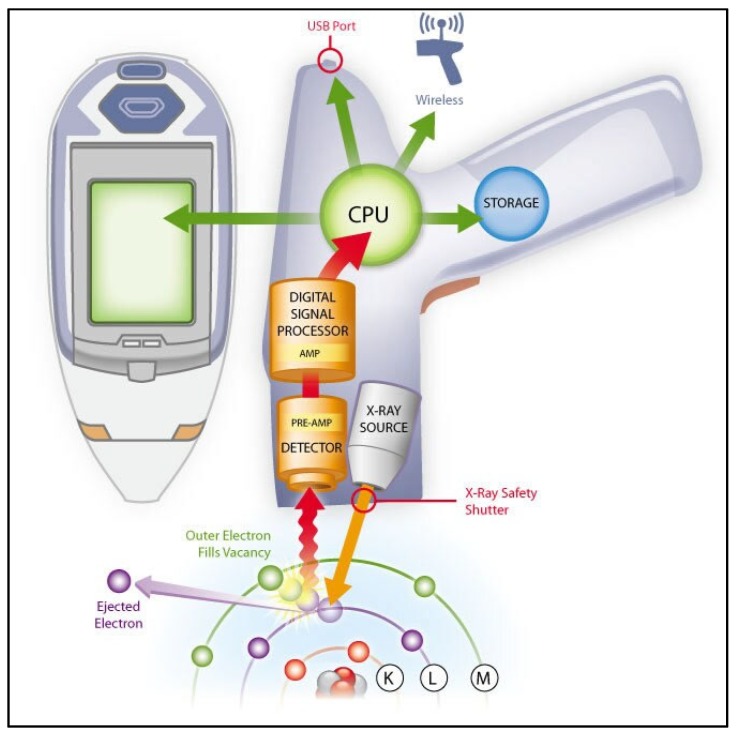
Components of a handheld XRF device for measuring the elemental composition [141].

X-ray fluorescence (XRF) is a method for determining the elemental composition of a sample in wt%. Compared to EDS, XRF is a non-destructive test and can be applied directly to the sample. Furthermore, because XRF operates without the need for a vacuum, liquid samples can be analyzed. In XRF, when an X-ray beam is concentrated on the sample, secondary (or fluorescent) X-rays will emit from the sample due to atom excitation as demonstrated. The high-energy X-ray source will cause dislodging of an electron from the inner orbital shell of an atom [136]. To fill in this vacancy, an electron will drop from another atom with a high-energy orbital shell [137]. This drop will cause the release of secondary or fluorescent X-ray. This emitted X-ray is unique for each element and this will be used for identification. XRF handheld devices are available and they are intensively used in the analysis of metals, soils, and food [138]. Figure 31 shows the components of a portable XRF device and electron excitation.

#### 5.2.1. Sample Preparation

XRF is a fast and non-destructive method for identifying and quantifying elements. The results are usually given in seconds. Most of the samples do not require preparation.

#### 5.2.2. Data Interpretation

**Figure 32:**
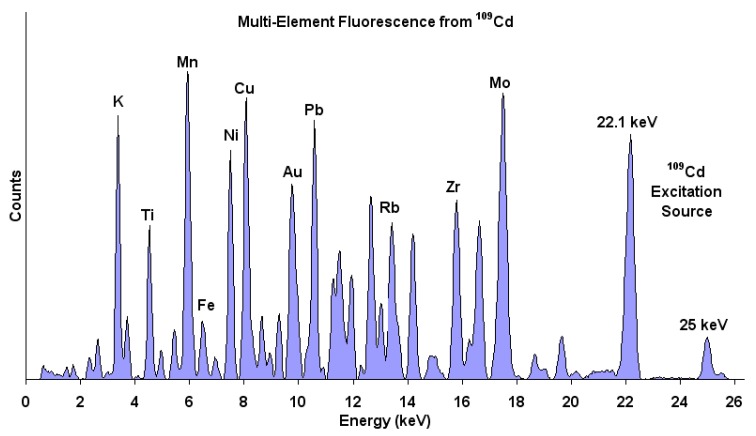
Identification of compounds using XRF by radioactive cadmium [139].

**Figure 33:**
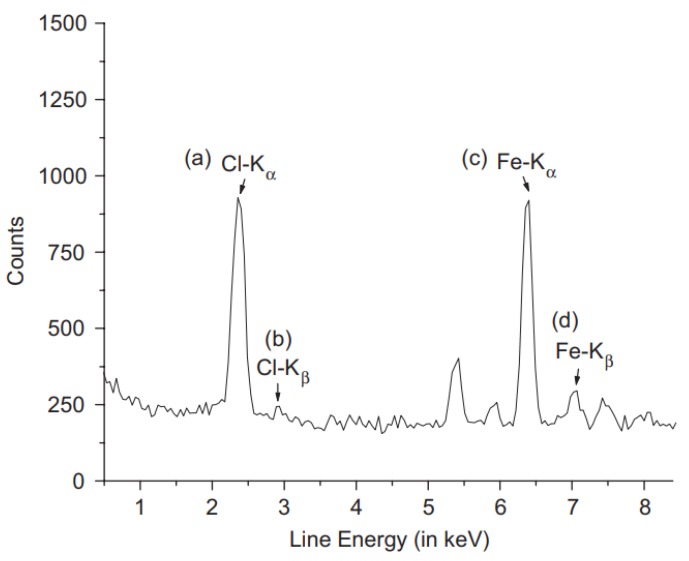
Detection of FeCl_3_ in a polycarbonate membrane for hydrogen separation [142].

XRF data are presented by plotting the intensity (counts) with energy (keV). Each element will have a unique energy peak for easier identification. Figure 32 shows some elements and their corresponding energy using cadmium as an excitation source. In addition, the relative amplitude of each peak can be used to calculate the mass percentage of the elements [139]. XRF was used to analyze a doped membrane made from polycarbonate and iron(III) chloride. The technique detected iron and chloride with the chemical formula of FeCl_3_ as given in Figure 33. Furthermore, a study was performed by XRF to determine the capability of trioctylmethylammonium thiosalicylate (TOMATS) membrane in extracting mercury from natural waters. The membranes were immersed inside the contaminated water and mercury was measured using in-situ XRF. The amounts of mercury was monitored with time to determine the removal efficiency [140]. Table 11 summarized the above studies for the use of XRF for membrane analysis.

#### 5.2.3. Limitations

XRF is a very useful tool for detecting the elements and their weight percentages. However, the quantification data will be given for the total element, not the ions. For example, if iron is measured, XRF will give the data for the total iron, not the mass percentage of each ion such as Fe^+2^ and Fe^+3^. This is because the difference in characteristic energy of ions is so small that the XRD detector cannot distinguish [143]. Moreover, some elements cannot be detected by XRF due to its weak fluorescent X-ray [142]. Some examples are hydrogen, carbon, and oxygen. However, polymers are organic compounds containing mainly hydrocarbons and therefore, and XRF cannot be used to analyze the pure polymer. Furthermore, the XRF beam penetrates deeply inside the sample for a few mm making it unsuitable for measurements of thin films of micrometers.

### 5.3. X-ray Photoelectron Spectroscopy

X-ray photoelectron spectroscopy (XPS) is a tool for identifying and quantifying the elements. XPS is considered as a sensitive surface-characterization technique as it gives the profile of the elemental composition along with the depth of 1 to 10 nm [48]. This is favorable for better and accurate measurements of the surface especially for thin-film membranes [144]. Furthermore, XPS can easily detect elements that EDS and XRF cannot detect.

**Figure 34:**
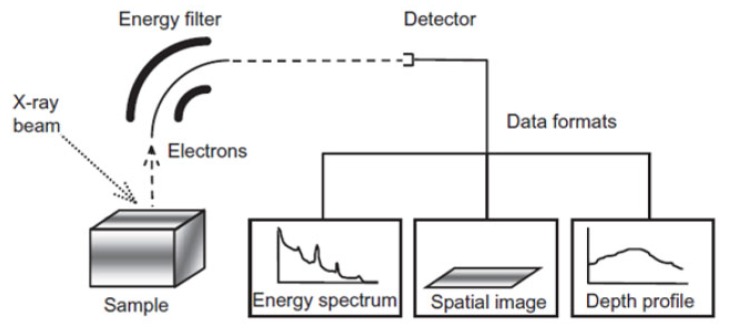
Components of X-ray photoelectron spectroscopy (XPS) for elemental analysis [149].

XPS works by sending a beam of X-ray to the sample and this will cause the release of electrons along with kinetic energy [145]. A detector is used to measure the number of electrons with the kinetic energy as demonstrated in Figure 34. Each element has a specific binding energy and this can be used to identify the element. XPS is also useful for determining the oxidation state of the element. For instance, sulfur compounds can have different forms such as sulfide (S^−2^), sulfite (SO3)^−2^, and sulfate (SO4)^−2^ and XPS can distinguish between these forms due to the difference in binding energy [146]. Furthermore, the detection limit in XPS is similar to EDS of 1000 ppm [147].

#### 5.3.1. Sample Preparation

XPS is a non-destructive test and usually does not require special preparation for polymeric membranes. Generally, the membrane is cut into small pieces with an area of 5 to 10 mm^2^ with sample depth not exceeding 4 mm to fit inside the chamber.

#### 5.3.2. Data Interpretation

**Figure 35:**
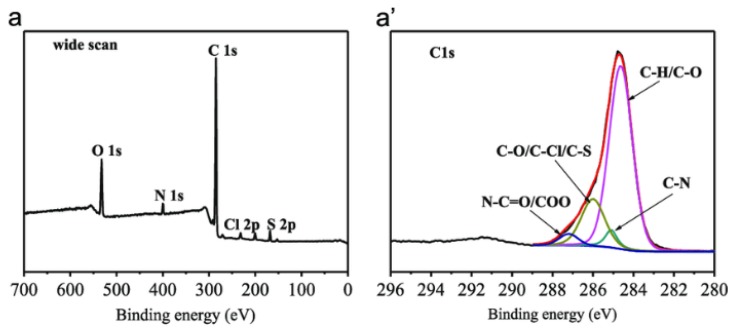
XPS analysis of: (**a**) Polyol-grafted polysulfone membranes, (**a’**) with the determination of carbon compounds [148].

The data in XPS are presented by the intensity (counts) and the binding energy (eV). First, a wide scan is performed to find the elements in the sample. After that, each element will be scanned separately to determine the functional groups and the oxidation state of the element. For instance, polyol-grafted polysulfone membrane was analyzed by XPS with a wide voltage range from 0 to 700 eV and five elements were detected such as oxygen, nitrogen, carbon, sulfur, and chlorine [148]. The carbon was again analyzed from 280 to 296 eV and a large single peak with asymmetrical shape was detected. This peak is formed due to the combination of other smaller peaks as shown in Figure 30. Different chemical classes were found such as ketones (C–O), alkanes (C–H), and amines (C–N) as demonstrated in Figure 35. Furthermore, the successful grafting of polyol on polysulfone was observed by the increase in O1s intensity and the reduction in C1s intensity [148].

XPS is also a very useful tool for studying the membrane degradation. During fuel cell operation, hydrogen peroxide is usually formed due to diffusion of oxygen across the membrane and its reaction with hydrogen [150]. Degradation of Nafion^®^ membrane was noticed by the decomposition of (CF_2_)*_n_* backbone to fluoride and sulfate ions [151].

Quantification of elements is generally performed in XPS based on the intensity peak [152]. The area under the curve is calculated then used to estimate the weight fraction of each element. This will also help in determining the chemical formula of the sample similar to EDS. Table 12 summarizes the discussed studies for membrane characterization by XPS.

#### 5.3.3. Limitations

XPS is an excellent technique for determining the oxidation state of the elements in a sample. Nevertheless, the analysis is only limited to solids due to the requirement of a high vacuum. XPS analysis is also time-consuming as it can take hours to cover a large area of the sample. Unfortunately, hydrogen and helium still cannot be recognized in XPS due to the absence of core electrons [153]. In addition, functional groups analysis by XPS could be difficult due to peaks overlap.

## 6. Conclusions

Characterization techniques are powerful tools for studying the physical structure and chemical properties of the membranes. These data are used thereafter for membrane development to enhance the performance. The basic characterization technique is the SEM where the membrane surface is examined. TEM provides a higher resolution compared to SEM for better studies on nanomaterials. One the other hand, AFM has a similar resolution to TEM but it can also provide some mechanical properties such as roughness. XRD is another tool for studying the crystal structure with the ability to name the compounds. SAXS and WAXS are more suitable for semi- and non-crystalline polymers with more statistics on the pore and particle sizes. FTIR, on the other hand, focuses on determining the functional groups, which can be used to identify the chemicals. Nevertheless, Raman spectroscopy is more sensitive in detecting these functional groups and requires almost no preparation. NMR spectroscopy provides another route for identifying the functional groups with minimum background interference. For the elemental analysis, EDS provides a basic elemental scan with a depth of 10 μm. XRF is a less sensitive technique for elemental analysis as the scan can reach up to 3 mm of depth, however the analysis can be directly applied by a handheld device with no sample preparation. On the other hand, XPS is a more advanced method for determining the elements and it is more of a surface technique due to the scanning depth of 10 nm. XPS can also give the weight composition along with the oxidation state of the elements.

It is clear that the selection of the characterization technique depends on the membrane nature and the required study. However, techniques such as SEM, XRD, and EDS are considered as fundamentals for membrane characterization. More advanced techniques can give additional information on the interfacial surface properties, molecular structure, and chemical properties. Comparison between the techniques in term of performed studies, advantages, and limitations is given Table 13.

## Figures and Tables

**Table 2:** Different studies performed by TEM for membrane characterization.

Study	Membrane	Methodology	Conclusion	Ref.
Agglomeration of particles in membranes	Polybenzimidazole (PBI) with titanium oxides particles	Cut samples in liquid nitrogen. Gold coated samples.	Low agglomeration effect	[31]
Film thickness	Polyacrylic acid (PAA)/polysulfone (PSf)	Stained samples by sodium hydroxide. Immersed samples in uranyl nitrate for 15 min. Washed samples with distilled water. Cut samples to 60-100 nm in thickness by ultramicrotome.	PAA film of 20 nm.	[29]

**Table 4:** XRD studies for characterization of polymeric membrane.

Study	Membrane	Methodology	Conclusion	Ref.
Membrane purity	Polyetherimide	Samples fractured in liquid nitrogen. Carbon tape to hold the samples.	Only polyetherimide peaks were detected indicating a pure sample.	[15]
Polymer Crystallinity	Polyethersulfone with aluminosilicate particles	Not reported.	Intensity increased indicating a more crystallized structure with better mechanical properties.	[66]
Polymer chain distance (*d*-space)	6H,12H-5,11-methanodibenzo[b,f][1,5]diazocine [PIM-EA(Me_2_)-TB]	Not reported.	Increase in *d*-space indicated transformation from glassy to rubbery phase.	[69]

**Table 5:** SAXS/WAXS studies for characterization of polymeric membrane.

Study	Membrane	Methodology	Conclusion	Ref.
Polymer-filler interaction and particle size measurements	Sulfonated poly(aryl ether ketone) (SPEEK) with phosphomolybdic acid (PMoA) filler	Fixed wavelength of 1.5Å. Vacuum operation at room temperature. Data calibrated using positron-emission tomography (PET).	No detection of –SO_3_H group indicated no nano-phase separation. Broad peak showed amorphous SPEEK structure. Particle radius of 524 Å.	[80]
Pore size measurements	Polymer of intrinsic microporosity (PIM) with amidoxime groups	Not reported.	Pore size distribution from 3.9 to 5.9 Å.	[78]

**Table 9:** Characterization of polymeric membranes by NMR.

Study	Membrane	Methodology	Conclusion	Ref.
Membrane purity	Polyetherimide	Spinning carbon (^13^C) to generate the magnetic field.	Only peaks of carboxylic acid, carboxylic-amide-carbon, phenyl-carbon-oxygen, and carbon-aromatic ring-amine were detected indicating a pure polyetherimide.	[19]
Polymer-filler interaction	Polysulfone with functionalized carbon nanotubes	Dissolved samples in deuterated chloroform.	Peaks of NH_2_ protons and amino-benzo-crown ether demonstrated the functionalization of carbon nanotubes in the polymer.	[121]
Polymer miscibility	Polysulfone and polyvinyl methyl ether(PVME)	Not reported	Increase in polymer intensity in the mixture indicated a good mixing.	[123]
Membrane degradation	Perfluorinated ionomer (Nafion^®^ 117)	Solid-state ^19^F NMR. Inserted samples in rotors filled with alcohol or water.	Detection of F^−^, SO_4_^−2^, and OH^−^ indicated membrane decomposition	[125]

**Table 10:** Characterization of polymeric membranes by EDS.

Study	Membrane	Methodology	Conclusion	Ref.
Membrane purity	Polyetherimide	Cut samples in liquid nitrogen. Coated samples by gold.	No additional elements to polyetherimide were detected indicating a pure sample.	[15]
Restoration of a fouled membrane by chemical cleaning	Polypiperazine-amide	Not reported	Reduction in sulfur content due to chemical cleaning showed membrane restoration.	[131]
Filler chemical formula	Poly(vinyl alcohol) and bismuth(III) oxide fillers	Not reported	The calculated formula matched bismuth(III) oxide.	[132]

**Table 11:** XRF studies for membrane characterization.

Study	Membrane	Methodology	Conclusion	Ref.
Chemical formula of filler	Polycarbonate and iron chloride filler	Not reported	Elemental composition of iron and chloride gives chemical formula of FeCl_3_ that matched the added filler.	[139]
Mercury extraction from natural waters	Trioctylmethylammonium thiosalicylate (TOMATS)	Cut sample to disks of 1 to 3 cm in diameter. Palladium target X-ray tube with beryllium window. SPECTRA EDX software for intensity measurements.	Mercury extraction by the membrane was monitored by detecting the amounts of mercury in the polymer.	[140]

**Table 12:** Studies for membrane characterization by XPS.

Study	Membrane	Methodology	Conclusion	Ref.
Surface chemical composition	polyol-grafted polysulfone	Monochromatic Al Kα X-ray source. Measurements at 45° take-off angle. Survey scan from 0 to 1000 eV then high-resolution scans of C1s regions.	O1s intensity increased while C1s intensity decreased confirming the grafting of hydroxyl groups on the membrane surface.	[148]
Membrane degradation	Nafion^®^ 112	Monochromator with Al Kα source. Step of 0.025 eV with 100ms.	Polymer backbone was decomposed due to detection of fluoride and sulfate ions.	[151]

**Table 13:** Comparison of various characterization techniques for analysis of polymeric membranes.

Technique	Performed studies	Advantages	Limitations
SEM	Surface topography. Pore size. Particle size Membrane Thickness	Magnification of up to 1 million.	Samples needs to be conductive. Not accurate for measurements less 10 nm.
TEM	Nanoparticles. Nanofilms. Nanopores. Membrane thickness.	Higher resolution than SEM.	Images does not show topography data. Staining the sample may be required. Thick samples (> 100μm) are difficult to be analyzed in TEM.
AFM	Nano-profiling. Surface roughness. Pore size distribution. Membrane stiffness.	No sample preparation. Similar resolution to TEM. Measurements of mechanical properties.	Requires more processing time. Lower depth of field.
XRD	Membrane purity. Compounds chemical formula. Crystal structure. Polymer chain distance.	No sample preparation. Detection of wide range of crystalline compounds.	Heavy elements are less sensitive to XRD. Less accuracy for small crystals. Peaks overlap for some compounds.
SAXS WAXS	Crystal structure. Polymer-filler interaction. Particle size distribution. Pore size measurements.	No sample preparation. Suitable for semi- and non-crystalline materials. More accurate average measurements for particle size and pore size.	Scattering intensity can be weak for some systems.
FTIR	Functional groups. Polymer-solvent compatibility. Polymer-filler interaction. Miscibility of polymer blends. Membrane degradation.	Detection of variety of compounds. High sensitivity of parts per million (ppm). Fast analysis time (in seconds).	Cannot analyze aqueous samples. Cannot detect molecules of two identical atoms.
Raman	Functional groups. Crystal structure. Polymer chain orientation. Polymer blends. Membrane fouling.	No sample preparation. More sensitive to functional groups with better intensity peaks.	Release of fluorescent light of some samples may cause background noise. Polar molecules have lower Raman signal.
NMR	Functional groups. Polymer-blend miscibility. Membrane decomposition.	Less background interference. Detection of polar molecules.	Liquid samples. Paramagnetic elements have less NMR signal.
EDS	Elemental composition. Chemical formula of fillers. Membrane fouling.	Fast analysis time.	Samples needs to be conductive. Limitation in detecting light elements. Cannot quantify ions.
XRF	Elemental composition. Chemical formula of fillers. Membrane degradation.	No sample preparation. In-situ analysis.	Very low sensitivity to hydrogen, carbon and oxygen. Cannot quantify ions. Unsuitable for thin film measurements.
XPS	Elemental composition. Functional groups. Formula of chemical compounds. Membrane degradation.	High sensitivity. Quantification of ions. Measurements of thin films of nm.	Solid samples. Cannot detect hydrogen and helium. Peaks Overlap for some elements. Long processing time.
